# Determining the Occurrence of Hypothyroidism Following Treatment With Radiation Therapy in Head and Neck Carcinoma Patients and the Associated Role of Risk Factors and Dose-Volume Histograms: A Prospective Study

**DOI:** 10.7759/cureus.31590

**Published:** 2022-11-16

**Authors:** Suparna K Pal, Saikat Das, Rajesh Isiah, Subhashini John

**Affiliations:** 1 Department of Radiation Therapy and Oncology, Institute of Post Graduate Medical Education & Research, Kolkata, IND; 2 Department of Radiation Oncology, All India Institute of Medical Sciences, Bhopal, Bhopal, IND; 3 Radiation Oncology, Christian Medical College, Vellore, Vellore, IND

**Keywords:** head and neck carcinoma, dose-volume histogram (dvh), imrt, quality of life, post-radiotherapy hypothyroidism

## Abstract

Background

Head and neck carcinomas are one of the most common malignancies in developing countries including India. Most patients are treated with radiotherapy. Although post-radiotherapy hypothyroidism is a known complication, data regarding its incidence and factors influencing it are scarce. This study aimed to determine the incidence of post-radiotherapy hypothyroidism in head and neck carcinoma patients treated with radiotherapy and the factors influencing it.

Methodology

Patients with head and neck carcinomas treated with radiotherapy as one of the modalities were included in this study. Thyroid function tests were done, and quality of life questionnaires were completed before treatment and during follow-up. Dose-volume histogram (DVH), demographic data, and disease-related parameters were compared.

Results

Out of the 95 patients screened, 14 were found to be hypothyroid prior to the commencement of radiotherapy and were excluded. With a median follow-up duration of 34 weeks, 29.6% developed hypothyroidism, with 19% developing it in the first year. On univariate and multivariate analysis of the DVH of the thyroid gland, volume receiving 50 Gy (V50), dose received to 50% volume (D50), and the mean dose (more than 50 Gy) were found to be significantly associated with hypothyroidism.

Conclusions

Hypothyroidism is a significant comorbid factor in Indian patients with head and neck carcinomas. The incidence of post-radiotherapy hypothyroidism is significant and occurs early compared to the western population leading to significant deterioration in the quality of life. Parameters such as the volume of the thyroid gland, V50, D50, and mean dose to the thyroid gland influence the incidence of hypothyroidism. The use of appropriate constraints can significantly prevent radiotherapy-induced hypothyroidism.

## Introduction

Head and neck malignancies are one of the most common malignancies in the world and in India [1]. A vast majority of them (about 78%) receive radiation therapy at one point or another [2]. Head and neck malignancies are known to have a very good response to concurrent chemoradiation or surgery followed by radiation. After complete treatment, it is known to be associated with longer overall survival. Therefore, the quality of life and long-term side effects are crucial. The global incidence of post-radiotherapy hypothyroidism in head and neck cancer patients varies from as low as 6% [3] to as high as 68% [4]. However, there is considerable debate regarding its incidence, influencing factors, relationship with dose, and the time of development. The available data for Indian patients, as reported by Aich et al., show an incidence of 0.57% at six weeks and 21.8% at two years [5]. A study by Murthy et al. [6] showed the incidence to be 51% at 41 months of median follow-up after treatment with intensity-modulated radiotherapy (IMRT) or three-dimensional conformal radiotherapy (3DCRT).

Biochemical investigations are available for the determination of hypothyroidism, and supplementation with exogenous thyroxin is possible. Hypothyroidism manifests as fatigue, poor appetite, and temper fluctuation, leading to an inability to meet social and familial roles [7]. It also affects the professional and economic productivity of the patients and their families. This adds to the financial strain that results from the cost of care and loss of workdays during the treatment period. Moreover, hypothyroidism results in long-term complications with respect to cardiac and general morbidity. Such morbidity in cancer survivors can be of immense physical, emotional, social, and economic problems. Thus, early detection and treatment of post-radiotherapy hypothyroidism can result in the avoidance of such complications and help improve the quality of life. Most guidelines, including the National Cancer Care Network (NCCN, USA) and the European Society of Medical Oncology (ESMO), have been developed based on western data. Their applicability to the Indian population is not well defined, especially in view of the heterogeneity of data, originating from different populations of the world. This difference is not only in the incidence but also in the time of occurrence of hypothyroidism post-radiotherapy. This study determines the incidence and risk factors associated with the development of hypothyroidism including the temporal association of the incidence in a population from the Indian subcontinent.

## Materials and methods

The study was cleared by the Institutional Review Board (IRB) and Ethics Committee of Christian Medical College, Vellore (approval number: 7003) and was registered with the Clinical Trials Registry of India (registration number: CTRI/2009/091/001007) before recruiting any patient.

Inclusion criteria

We included adult patients with non-metastatic head and neck carcinomas, with an Eastern Cooperative Oncology Group (ECOG) performance status of -0 to 2, and receiving more than 45 Gy as part of treatment with external-beam radiotherapy (EBRT), with radiation field extending to the unilateral or bilateral lower neck.

Exclusion criteria

We excluded patients with any present or past history of hypothyroidism, with a history of previous exposure to radiation therapy in the head and neck region, and those with clinical or radiological intracranial extension.

Sample size

Considering a likely incidence of hypothyroidism in this cohort to be 20% (±10%), the estimated sample size was calculated to be 64. Correcting for anticipated attrition (beta error) due to loss of follow-up or death of about 20% of the patients, the target sample size was recalculated to be 80.

Methods

The patients were explained about the study. Informed consent was obtained from the willing patients in the IRB Ethics Committee-approved Informed Consent Doccument (ICD), and were then included for screening. Screening comprised blood levels of thyroid-stimulating hormone (TSH), free thyroxine (FTC), and tetraiodothyronine (T4). In addition, the thyroid questionnaire and the Functional Assessment of Cancer Therapy Quality of Life Head & Neck (FACT QOL H&N) questionnaire were utilized. The tests were then repeated at every follow-up post-radiotherapy.

Patients meeting the inclusion criteria were included in the study. All included patients underwent the required treatment as per the institutional protocol for their diagnosis, staging, histology, general condition, and treating oncologist’s decision.

Statistical analysis

The results obtained were analyzed for the incidence of hypothyroidism, as well as the demographic and treatment factors influencing it. The chi-square test was run for categorical variables, while univariate and multivariate analyses were done for the dose-volume histogram (DVH) parameters. Wilcoxon signed-rank tests were used for comparing the quality of life data. SPSS version 21 (IBM Corp., Armonk, NY, USA) and GraphPad (GraphPad Software, Inc., San Diego, CA, USA) quick-cals were used as statistical software.

The initial results were submitted to The Tamil Nadu Dr MGR Medical University in 2011 for partial fulfilment of the MD (Radiation Oncology) degree and are included in the University repository [8]. The present submitted study is the long-term follow-up of the study with updated results and analysis.

## Results

Of the 95 patients who were screened, 14.7% (14) were found to be hypothyroid at presentation. Of the 81 patients included in the study, two died and eight were lost to follow-up. The duration of follow-up ranged from one week to 129 weeks, with a median follow-up duration of 34 weeks and a mean follow-up duration of 39 weeks. Thus, 71 patients were included in the present analysis.

Demography

Most patients in this cohort (80.3%) were males in keeping with the epidemiological trends of head and neck carcinomas in the Indian subcontinent. Nine patients had hypertension and three had diabetes mellitus. Moreover, eight patients were from the Himalayan or sub-Himalayan region which is known to be an endemic region for hypothyroidism. Nearly 50% (35) of the patients had a history of smoking or chewing tobacco and beetle nuts or both.

Disease characteristics

Most patients had a primary in the larynx (35.2%), oral cavity (19.7%), oropharynx, and hypopharynx (15.5% each).

Outcomes

Among the 71 patients, the incidence of hypothyroidism was 29.6% (21) during the follow-up period. In total, six patients developed hypothyroidism before three months and nine patients before six months from the date of completion of radiotherapy. Additionally, 14 patients developed hypothyroidism within the first year of radiotherapy.

The primary site, gender of the patient, and the beam used in the treatment (cobalt 60/6 MV photons) did not show any defining role.

DVH was available for 38 patients who were treated with IMRT on a Clinac machine (Varian Medical Systems, Palo Alto, CA, USA). The volume of the thyroid varied from 4.9 cc to 30 cc, with a median volume of 11.37 cc. The maximum dose received to a point varied from 4,810 cGy to 7,597 cGy, and the mean dose varied from 596 cGy to 6,827 cGy.

On univariate and multivariate analysis of the DVH of the thyroid gland, volume receiving 50 Gy (V50), dose received to 50% volume D50), and the mean dose were found to be significantly associated with hypothyroidism. V10, V20, V30, V40, V45, V60, and the maximum point dose were not found to be significant (Table 1).

**Table 1:** Test of significance (p-value) of dose-volume histogram parameters.

Parameter	Univariate	Multivariate
V10	0.07	0.31
V20	0.02	0.19
V30	0.01	0.13
V40	0.009	0.11
V50	0.007	0.04*
Mean dose	0.007	0.05*
D50	0.004	0.05*

## Discussion

Hypothyroidism is a common side effect after radiotherapy to the head and neck region. The most common diseases requiring the same are head and neck malignancies. The outcomes of such treatment in early and locally advanced malignancies are not as dismal as it was in the past. Thus, the need for long-term quality of life needs to be asserted. According to studies conducted in the west, the onset of hypothyroidism is mostly late, and the recommendations for its monitoring are varied. While the NCCN recommends monitoring every 6-12 months beginning at six months, the ESMO recommends monitoring at one, two, and five years. The European Society of Therapeutic Radiation Oncology/Tata Memorial Hospital international symposium in 2005 [9] recommended monitoring T3, T4, and TSH annually, particularly in patients with laryngeal malignancies.

In India, the incidence of hypothyroidism in the general population is quite significant. According to a study by Shantha et al. [10] conducted in the urban area of Chennai, which is expected to have a low incidence of hypothyroidism due to its proximity to the sea and dietary patterns, the incidence of hypothyroidism was 8.8%. The subclinical hypothyroidism in iodine-sufficient patients in India was found to be 9.8% in a study by Unnikrishnan et al. conducted in Cochin [11]. In Delhi, the incidence was far greater, with 19.3% of the population having subclinical and 9.6% having clinical hypothyroidism, as documented by Marwaha et al. [12]. Our study showed an incidence of 14.7% in the initial patient cohort comprising patients from both the southern coastal belt as well as the sub-Himalayan belt including north-east India.

Post-treatment hypothyroidism in the present cohort was 29.6% at a median follow-up time of 39 weeks. The incidence of post-radiotherapy hypothyroidism in the literature varies widely. With regards to prospective Indian data, Aich et al. [5] recorded an incidence of 21.8% at two years (mainly from eastern India), Nirmala et al. [13] 42.2% at nine months (Karnataka and other southern states), and Laway et al. [14] 16.94% at 2 years (Jammu and Kashmir). However, all of these studies were done on patients who had received conventional EBRT, mostly with cobalt 60 beams. The only data on 3DCRT and IMRT patients in India are analyses of data from two simultaneously randomized studies by Murthy et al. [6] which showed an incidence of 51% at 41 months of median follow-up. However, these studies were not specifically designed to look into radiation-induced hypothyroidism. Our cohort contained a mix of conventional EBRT delivered with cobalt 60 or 6 MV photons, 3DCRT with 6 MV photons, and IMRT with 6 MV photons.

Most of the initial western literature studied hypothyroidism as a late effect. However, Alkan et al. [15] showed an average duration of onset of 6.08 months and 83% of the patients developed hypothyroidism within nine months. Nishiyama et al. [16] described a significant rise at four and six months from the start of radiotherapy (~4.5 months from completion of radiotherapy). Sinard et al. [17] found the average time of development of hypothyroidism to be about 8.2 months (with a range of 1-21 months). Indian literature too, as mentioned, did show a trend toward the early onset of hypothyroidism compared to the early western literature. Our present cohort had a follow-up duration of up to 129 weeks with a median of 34 weeks and a mean duration of 39.7 weeks. A Kaplan-Meir curve was generated with the available data and after censoring the data showed an estimated incidence of 30% at one year and 55% at two years from the date of completion of radiotherapy.

DVH and its implications

As reported by Emami et al. [18], tolerance values of 8/5, 13/5, and 35/5, i.e., the incidence of clinical hypothyroidism, respectively, in 8%, 13%, and 35% of patients after five years of radiation therapy when the dose received were 45 Gy, 60 Gy, and 70 Gy, respectively. However, as the thyroid gland is composed of multiple independent functional subunits, it is a parallel organ where not only the maximum point dose but both dose and volume are expected to play a significant role in determining the outcome. Therefore, this data is valid only when the entire thyroid gland is within the field of radiation and is uniformly irradiated, which is not the case in most modern conformal radiation and IMRT.

Alkan et al. [14] reported that the dose received by the entire thyroid gland is a significant risk factor. The dose received to the entire thyroid gland in their study was 6.07 ± 3.5 Gy in patients who had developed hypothyroidism and was significantly higher than the group who remained euthyroid at 5.92 ± 2.5 Gy (p < 0.05). Bhandare et al. [19] noted that if the treated volume was ≥85% then the chance of developing clinical hypothyroidism was 23% compared to 0% when the treated volume was <50%. The incidence of subclinical hypothyroidism was 25% and 10% for bilateral and unilateral neck node irradiation, respectively.

Using DVH, Alterio et al. [20] did not find any relationship between the irradiated thyroid volume and dose with respect to V10, V30, and V50, where V10, V30, and V50 are thyroid volumes irradiated by a dose of over 10, 30, and 50 Gy, respectively. However, the scope of use of DVH in their study was limited by the fact that the study cohort had a relatively uniform DVH.

Yoden et al. [21] in their study found V10-60 (percentage of the volume of thyroid gland receiving radiation doses between 10 and 60 Gy) to be a possible risk factor. They also reported that V30 (percentage of volume receiving >30 Gy) was a predictor of peripheral hypothyroidism. It was also found to be significantly correlated with peak serum TSH levels.

A prospective study examining the role of DVH in head and neck carcinoma patients by Boomsma et al. [22] showed higher mean thyroid gland dose and decreased thyroid gland volume was the best fit for the Normal Tissue Complication Probability (NTCP) model of radiation-induced hypothyroidism. Cella et al. [23] showed in a similar retrospective study in Hodgkin disease patients that female sex and absolute thyroid gland exceeding 30 Gy were the main factors corresponding to an NTCP model.

In a study of dosimetric analysis, Akgun et al. [24] found that V30, thyroid volume, and Dmean were correlated to hypothyroidism in univariate analysis but none in multivariate analysis. In another study, Kim et al. demonstrated that V35-V50 was significantly associated with hypothyroidism in univariate analysis, while only V45 was found to be associated in multivariate analysis [25].

In our study conducted among Indian patients, the volume of the thyroid gland, the volume of the thyroid receiving 50 Gy, the mean dose received to the thyroid gland, and the dose received to 50% of thyroid volume were found to be statistically significant in univariate and multivariate analysis. The other Indian data regarding the dose-volume parameters and hypothyroidism by Murthy et al. [6] showed that high doses per fraction and D100 were significantly associated with the development of hypothyroidism.

A recent Danish Head and Neck Cancer Study Group (DAHANCA) 2013 guidelines for IMRT for head and neck cancers suggests giving an optional constraint of the mean dose to the thyroid gland to less than 40 Gy wherever feasible [26]. However, this value is based on limited available data.

**Figure 1:**
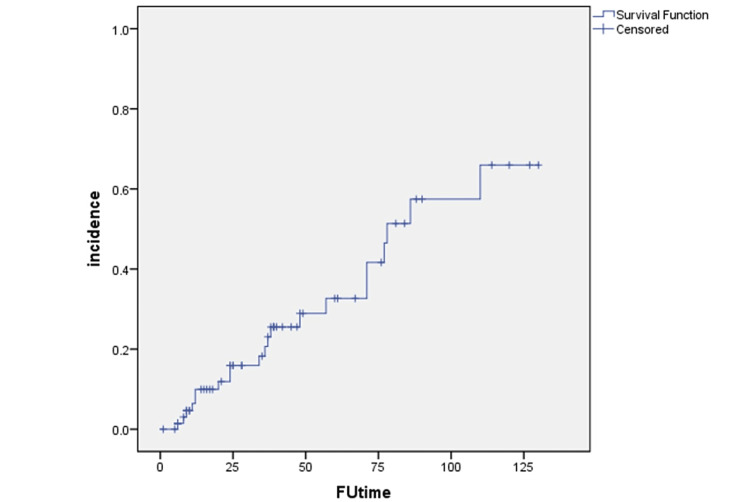
Kaplan-Meier curve for the incidence of hypothyroidism. FUtime: follow-up time in weeks

In our cohort, all patients developing hypothyroidism had received a mean dose of more than 50 Gy. The mean dose in those patients ranged from 50.28 to 68.27 Gy, with a mean value of 59.34 Gy compared to the cohort of patients who retained normal thyroid function with a range of 5.69 to 67.89 Gy, with a mean value of 47.86 Gy (Figure 1).

Quality of life

The quality of life was estimated by the FACT score [27]. The data collected were analyzed and the total FACT scores had a significant improvement from the baseline when tested with Wilcoxon signed-rank test.

However, the FACT score for those with hypothyroidism did not show any difference from the baseline value when compared to that at the point of developing hypothyroidism (p = 0.068), suggesting that the improvement in quality of life post-radiotherapy was not significant when patients developed hypothyroidism.

For most Indian cancer survivors, the quality of life in the form of functionality, acceptability in the family and society, and economic productivity remain key issues.

## Conclusions

The present prospective study on Indian patients shows that hypothyroidism is a significant comorbid factor in patients with head and neck carcinomas. The incidence of post-radiotherapy hypothyroidism is significant and occurs early compared to early western reports. The incidence of hypothyroidism significantly decreases the quality of life of cancer survivors. Thyroxin supplements that are available at a low cost can prevent the deterioration of the quality of life. Regular biochemical blood tests can lead to early detection and treatment. The volume of the thyroid gland, V50, D50, and mean dose to the thyroid gland are associated with the incidence of hypothyroidism and may be considered for comparing the risk of hypothyroidism in competing IMRT plans. Further prospective trials for the determination of appropriate constraints based on the mean thyroid dose, V50, and D50 are warranted in the present era of IMRT and image-guided radiotherapy for the prevention of radiation-induced hypothyroidism.
